# Key interventions and outcomes in perioperative care pathways in emergency laparotomy: a systematic review

**DOI:** 10.1186/s13017-025-00597-4

**Published:** 2025-03-10

**Authors:** Deena P. Harji, Ben Griffiths, Deborah Stocken, Rupert Pearse, Jane Blazeby, Julia M. Brown

**Affiliations:** 1 Manchester University NHS Foundation Trust, Manchester, UK; 2 Clinical Trials Research Unit, Leeds Institute of Clinical Trials Research, University of Leeds, Leeds, UK; 3 Faculty of Medicine and Dentistry, Queen Mary University of London, London, UK; 4 Bristol Centre for Surgical Research, Population Health Sciences, University of Bristol, Bristol, UK; 5 NIHR Bristol Biomedical research centre, Bristol, UK

**Keywords:** Emergency laparotomy, Pathways, Emergency surgery

## Abstract

**Introduction:**

Emergency laparotomy (EmLap) is a complex clinical arena, delivering time-sensitive, definitive care to a high-risk patient cohort, with significant rates of post-operative morbidity and mortality. Embedding perioperative care pathways within this complex setting has the potential to improve post-operative outcomes, however, requires an in-depth understanding of their design, delivery and outcome assessment. Delivering and implementing complex interventions such as perioperative pathways require transparent reporting with detailed and indepth description of all components during the assessment and evaluation phase. The aim of this systematic review was to identify the current design and reporting of perioperative pathways in the EmLap setting.

**Methods:**

The OVID SP versions of MEDLINE, EMBASE and the Cochrane Central Register of Controlled Trials were searched between January 1950 and December 2023. All randomised and non-randomised cohort studies reporting outcomes on perioperative care pathways in adult patients (> 18 years old) undergoing major emergency abdominal surgery were included. A narrative description of all perioperative pathways included was reported to identify design and description of the pathway including the delivery and timing of component interventions. All pathways were evaluated against the Template for Intervention Description and Replication (TIDieR) checklist.

**Results:**

Eleven RCTs and 19 non-randomised studies were identified, with most studies considered to be at moderate risk of bias. Twenty-six unique pathways were identified and described, delivering a total of 400 component interventions across 44,055 patients. Component interventions were classified into 24 domains across the perioperative pathway. Twenty studies (66.6%) did not report the TIDieR framework items, with thirteen studies reporting less than 50% of all items. Two hundred and fifty individual outcomes were reported across pathways, with the most commonly reported outcomes related to morbidity, mortality and length of stay.

**Conclusion:**

Current perioperative pathways in EmLap setting are underpinned by variable component interventions, with a lack of in-depth intervention reporting and evaluation. Future studies should incorporate the TIDieR checklist when reporting on perioperative pathways in the EmLap setting.

**Clinical trial number:**

Not applicable.

## Background

Major emergency abdominal surgery is a complex clinical arena serving a heterogenous patient population, with variable physiological status. This high-risk cohort requires time-sensitive, definitive care to potentially mitigate the impact of their physiological and pathological status on post-operative outcomes. The burden of emergency surgery is significant, with reported rates of post-operative morbidity and mortality of 14–47% and 10–20% respectively [1, 2]. There have been considerable efforts made in recent times to try and improve these outcomes through the introduction of structured and standardised care pathways to attenuate the physiological stress of emergency laparotomy and improve post-operative clinical outcomes. Initiatives such as the ELPQuiC (Emergency Laparotomy Quality Improvement Care Bundle) have demonstrated the feasibility of implementing dedicated EmLap pathways into the early peri-operative period in the emergency setting to improve post-operative mortality [3–5]. Modified Enhanced Recovery after Surgery (ERAS) protocols in the emergency setting have demonstrated improvements in broader clinical outcomes, including reduced length of stay, post-operative complications and improved gastrointestinal functions [6, 7].

These perioperative pathways often comprise several components, which interact to exert their overall effects. As demonstrated by the EPOCH trial, it is the combination of high-fidelity component interventions and overall compliance to the perioperative pathway, that drives overall improvement [8]. Understanding the design and delivery of perioperative pathways in the EmLap setting is essential to evaluate their clinical and cost-effectiveness, and to facilitate broader adoption and implementation. Surgical and perioperative interventions are often poorly reported with a lack of detailed and in-depth intervention reporting [9–12]. There is growing recognition of the importance of intervention reporting. The Template for Intervention Description and Replication (TIDieR) checklist and guide was developed in 2014 to provide a structure for assessing the completeness of intervention descriptions [13]. The overarching purpose of the TIDieR checklist is to describe interventions in sufficient detail to allow their replication. The use of the TIDieR checklist has led to enhanced and in-depth reporting of complex interventions, which has led to improved implementation across clinical practice and trials [14–16]. Detailed reporting of the types of interventions delivered across EmLap perioperative pathways, as well as, key aspects of each component, including mode of delivery, frequency, intensity and overall duration, is essential to ensure effective and time sensitive treatment is delivered. Comprehensive reporting of all aspects of perioperative pathways is important in clinical studies to ensure appropriate assessment of clinical effectiveness and onward implementation into clinical practice. Incorrect implementation leads to the initiation of ineffective or lesser treatment. This has implications for the patient, potentially impacting on their clinical outcomes, and on wider healthcare resources.

The aim of this systematic review was to identify the current design and make-up of perioperative pathways in the EmLap setting, including identifying component interventions, their associated reported clinical and patient-reported outcomes and to understand their design and reporting in line with the TIDieR checklist.

## Methods

This systematic review was conducted according to a pre-specified protocol based on guidance from the Centre for Reviews and Dissemination [17] and the Cochrane Handbook [18] and is reported in line with the PRISMA (Preferred Reporting Items for Systematic Reviews and Meta-Analyses) statement [19]. Our protocol was registered with the international, prospective register of systematic reviews, PROSPERO (CRD42021277211).

### Eligibility criteria

All randomised and non-randomised cohort studies reporting outcomes on perioperative care pathways (PCP) in adult patients (≥ 18 years old) undergoing major emergency abdominal surgery were included. Perioperative care pathways were defined as multimodal perioperative care bundles, perioperative protocols, dedicated clinical pathways or ERAS protocols comprising of a number of components. Studies were excluded if they reported on perioperative care protocols/pathways in the trauma or elective setting or did not include clinical outcomes.

### Search strategy

The OVID SP versions of MEDLINE (1950 to 31st December 2023), EMBASE (1980 to 31st December 2023) and the Cochrane Central Register of Controlled Trials were searched using the following search terms ‘emergency surgery’, ‘laparotomy’ ‘enhanced recovery’, ‘fast track’, ‘multimodal’, ‘care bundles’, ‘perioperative protocols’, ‘care pathways’ separated by the Boolean operator ‘AND’. Reference lists of included articles were hand-searched to identify any additional studies. All citations were collated within EndNote X7.8^®^, USA and duplicates were removed. All relevant titles and abstracts were screened by two reviewers (DH and BG). The full text versions of potentially eligible abstracts were retrieved in full. Only studies that fulfilled all eligibility criteria were included. Any conflicts were resolved through discussion.

### Study quality

Methodological quality assessment of included studies was undertaken using the ‘Risk of Bias In Non-Randomised Studies of Intervention’ (ROBINS-I) assessment tool [20] for non-randomised studies and the Cochrane risk of bias tool for randomised controlled trials (RCTs) [21].

### Data analysis

A narrative description of all perioperative pathways was reported to identify design of the pathway including the delivery and timing of component interventions. To assess the completeness of intervention reporting and its replicability each PCP was assessed against the TIDieR checklist. To assess the consistency of outcome reporting the frequency of each definition and any inconsistencies in definitions across individual studies were reported. Descriptive data were expressed using basic statistics including proportions and averages. All data were entered into Microsoft Excel (Microsoft, Redmond, Washington USA) for analysis.

## Results

**Fig. 1:**
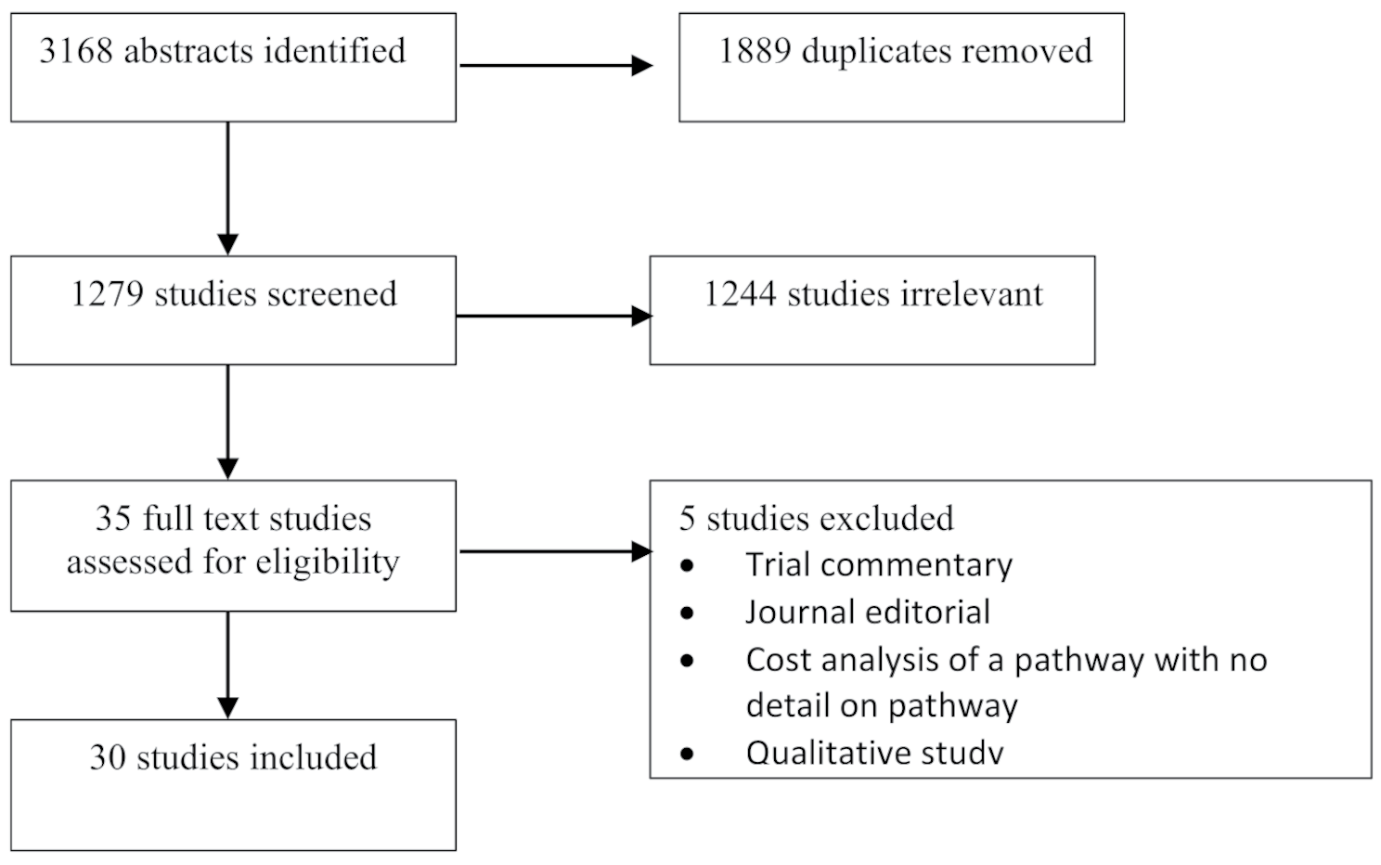
Search Strategy

A total of thirty studies outlining 26 unique pathways in EmLap were included in this review [3, 5, 8, 22–48]. A total of 10 randomised controlled trials (RCTs), 1 pilot RCT, 4 prospective cohort studies, 1 propensity matched cohort study, 5 retrospective cohort studies, 8 before and after studies and 1 case-control study were included (Table 1; Fig. 1). Outcomes were reported in 44,055 patients undergoing major emergency abdominal surgery. Care pathways were defined in different ways with 16 studies reporting on emergency ERAS protocols, 7 studies reporting on care bundles, 3 studies reporting on the implementation of a perioperative protocol, 2 studies reporting on protocolised care pathways and 1 study defined its care pathway as intermediate care and 1 study defined it PCP as a quality improvement programme. The earliest reported perioperative pathway was in 2011, with a total of 3 studies predating the introduction of the TIDieR checklist, and 27 studies published following its introduction.

**Table 1:** Studies included

Author	Year	Year Included	Country	Study Type	Protocolised Care Pathway	Patient Population	Total No of Patients
Moller	2011	2008–2009	Denmark	Before and After Study	Perioperative protocol	Perforated Peptic Ulcer	627
Gonenc	2014	2012–2013	Turkey	Single centre RCT	ERAS	Laparoscopic Perforated Duodenal Ulcer	47
Huddart	2014	2012–2013	UK	Before and After Study	Care bundle	Emergency abdominal surgery	726
Lohsiriwat	2014	2011–2013	Thailand	Retrospective cohort	ERAS	Obstructed colorectal cancer	60
Vester-Andersen	2015	2010–2012	Denmark	Multicentre RCT	Intermediate Care	Emergency abdominal surgery	286
Wisley	2015	2008–2012	Australia	Retrospective cohort	ERAS	Emergency abdominal surgery	370
Tengburg	2017	-	Denmark	Case-control study	Perioperative protocol	Emergency abdominal surgery	1200
Ebm	2018	2012–2013	UK	Before and After Study	Care bundle	Emergency abdominal surgery	726
Mohsina	2018	2014–2016	India	Single centre RCT	ERAS	Perforated Duodenal Ulcer	102
Shang	2018	2010–2017	China	Propensity matched cohort study	ERAS	Obstructed colonic cancer	636
Aggarwal	2019	2016–2017	UK	Before and After Study	Care bundle	Emergency abdominal surgery	14,809
Burcharth	2019	2017–2018	Denmark	Prospective cohort	Perioperative protocol	Emergency abdominal surgery	227
Doyle	2019	2012–2013	UK	Before and After Study	Care bundle	Emergency abdominal surgery	716
Liska	2019	2014–2017	USA	Retrospective cohort	ERAS	Patients were included in the study if they underwent colorectal surgery during a nonelective admission, defined as admission from the emergency room or as transfer from an outside hospital (OSH).	404
Lohsiriwat	2019	2011–2017	Thailand	Prospective cohort	ERAS	Emergency colorectal surgery	60
Jordan	2020	2014–2019	UK	Before and After Study	Care bundle	Emergency abdominal surgery	930
Peden	2020	2014–2015	UK	Multicentre RCT	Quality improvement intervention	Emergency abdominal surgery	15,873
Saurabh	2020	2017–2018	India	Single centre RCT	ERAS	Small bowel pathology	82
Vinas	2020	2011–2017	Spain	Prospective cohort	ERAS	Left colonic perforation	50
Fischer	2020	2017–2019	USA	Retrospective cohort	ERAS	Emergency colorectal surgery	3086
Ong	2021	2017–2019	Singapore	Before and After Study	Perioperative care pathway	Emergency abdominal surgery	314
Sharma	2021	2019–2020	India	Single centre RCT	ERAS	intestinal perforation and small bowel obstruction	100
Masood	2021	2018–2019	Pakistan	Single centre RCT	ERAS	Perforated Duodenal Ulcer	42
Pranavi	2022	2018–2020	India	Single centre RCT	ERAS	Patients with perforation peritonitis on the basis of examination and imaging, and planned for laparotomy in the emergency setting	120
Boden	2022	2015–2016	Australia	Single centre Pilot RCT	Protocolised care pathway	Emergency abdominal surgery	50
Ceresoli	2023	2020–2021	Italy	Prospective cohort	ERAS	Intestinal resections with or without anastomosis, hollow viscus injury repair, enteric bypass or adhesiolysis	589
Aggarwal	2023	2021–2022	India	Single centre RCT	ERAS	Acute intestinal obstruction	60
Timan	2023	2018–2023	Sweden	Before and After Study	Protocolised care pathway	Emergency abdominal surgery	1344
Pathrikar	2023	2021–2022	India	Single centre RCT	ERAS	Perforated Duodenal Ulcer	41
Trangbæk	2023	2015–2019	Denmark	Retrospective cohort	Perioperative care bundle	Emergency abdominal surgery	378

### Study Bias

**Fig. 2:**
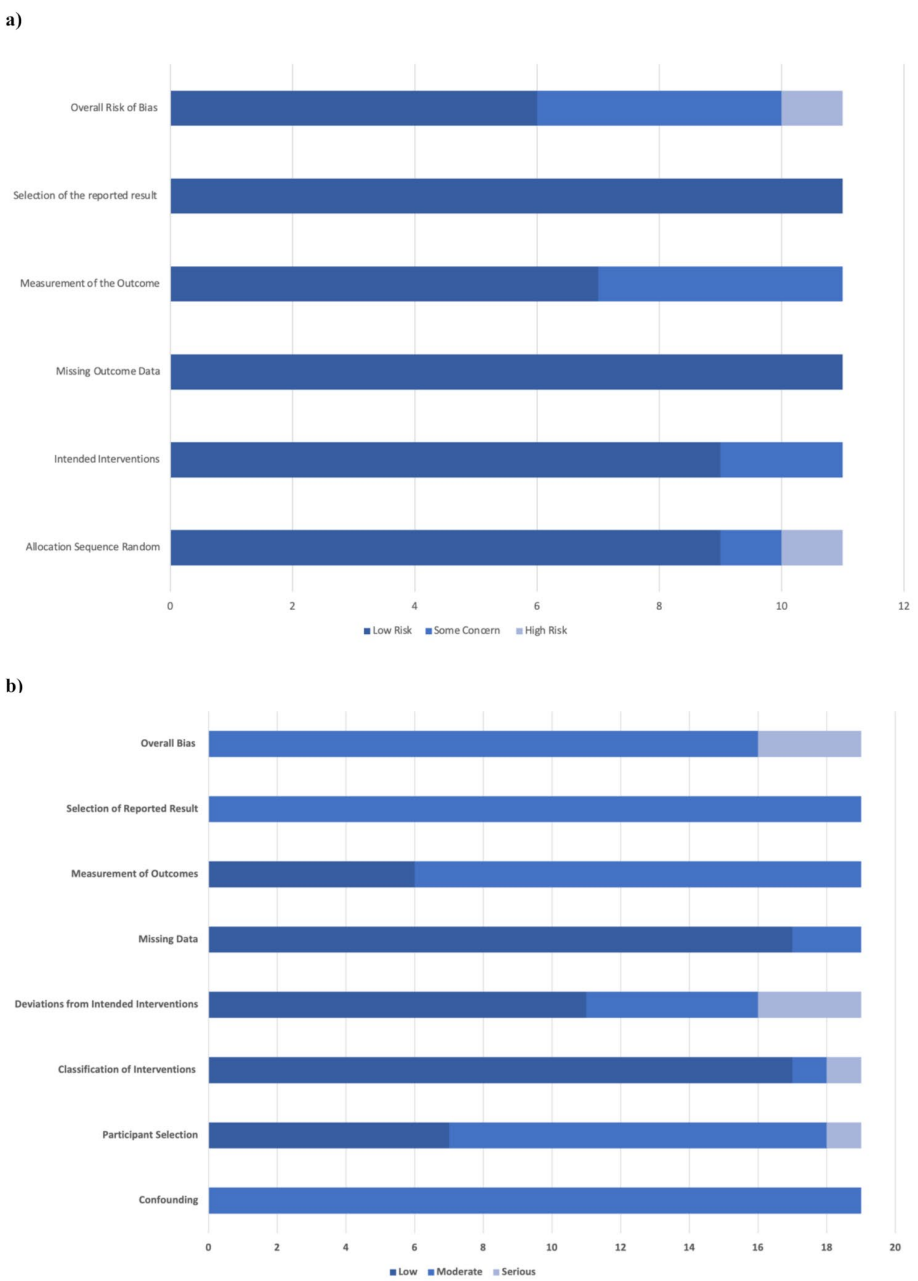
(**a**) Risk of Bias Summary for RCTs. (**b**) Risk of Bias Summary for non-randomised studies

The majority of RCTs were low overall risk of bias: with 6 RCTs identified to be low risk, 4 RCTs were considered to have some concerns and 1 RCT was considered to be high risk (Fig. 2a). The majority of 19 non-randomised studies were moderately biased: with 16 identified moderate risk and 3 considered to be seriously biased (Fig. 2b). Key areas for concern include confounding variables, participant selection, measurement of outcomes and selection of reported results.

### Peri-operative pathway design

**Fig. 3:**
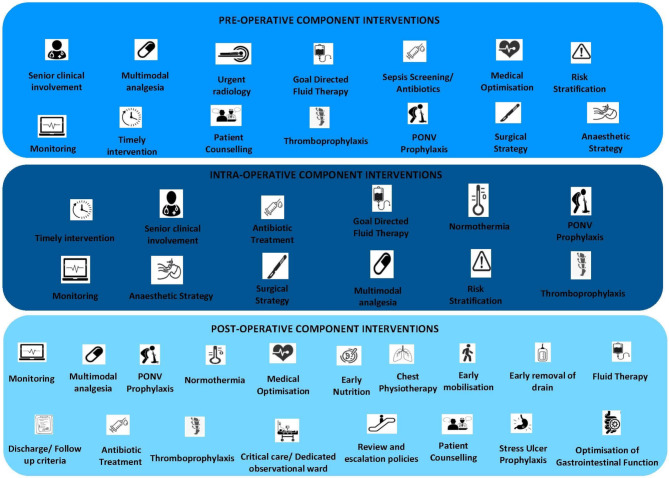
Component Interventions

Twenty-six unique pathways were identified, with a total of 400 component interventions delivered across all studies. These component interventions were classified into 24 domains (Fig. 3) across three distinct time points; pre-, intra- and post-operatively. There was significant overlap with delivery of domain interventions across perioperative timepoints. Six domains; multimodal analgesia, goal-directed fluid therapy, antibiotics, monitoring, thromboembolism prophylaxis and post-operative nausea and vomiting (PONV), were delivered across all three timepoints. Urgent radiology was identified as the only domain intervention delivered exclusively in the pre-operative phase. Risk stratification, timely intervention, prescriptive anaesthetic strategy and prescriptive surgical strategy were domain interventions delivered during the pre- and intra-operative phases of PCPs. There were no exclusive intra-operative domain interventions identified. Five domains were exclusively delivered during post-operative phase; early nutrition, chest physiotherapy, early mobilisation, early removal of drains and discharge/follow up criteria. Three domain interventions were delivered in the pre- and post-operative phases: medical optimisation, review and escalation policies and stress ulceration prophylaxis. Maintaining normothermia was the only domain that was delivered in the intra- and post-operative phases.

Twenty-one studies reported on EmLap care pathways with a pre-operative phase, consisting of a median of 6 individual components (Table 2). A total of 108 pre-operative component interventions were mapped to 14 broad pre-operative intervention domains. There was significant variation in the coverage of domains delivered in the pre-operative phase, with the sepsis screening/antibiotic prophylaxis domain being the most commonly employed; 14 (66.7%) studies reported component interventions within this domain.

**Table 2:** Pre-operative intervention domains

Author	Number of component interventions	Senior clinical involvement	Non-opioid multimodal analgesia	Urgent radiology	Goal directed fluid resuscitation	Sepsis screening /Antibiotic prophylaxis	Pre-operative medical optimisation	Monitoring	Risk Stratification	Timely Intervention	Patient Counselling	Thromboembolism prophylaxis	PONV Prophylaxis	Surgical Strategy	Anaesthetic Strategy
Moller	12	*				*	*	*	*	*					
Huddart	5				*	*		*		*					
Lohsiriwat	1										*				
Gonenc	6		*		*	*					*		*		
Wisley	4						*					*			
Tengburg	6	*		*	*	*			*	*					
Shang	6					*					*	*	*		
Mohsina	1		*												
Burcharth	6			*		*	*			*					
Liska	4		*			*						*	*		
Lohsiriwat (2019)	3						*				*			*	*
Aggarwal	2				*						*				
Peden	10	*	*		*	*	*		*	*	*				
Saurabh	2		*												
Vinas	6					*	*					*			
Ong	6	*				*			*						
Sharma	6				*	*					*			*	
Pranavi	3		*										*		*
Timan	7	*			*	*		*		*					
Ceresoli	5					*			*						*
Trangbæk	7	*		*		*								*	
**Domain Coverage**	**108**	**28.6**	**28.6**	**14.3**	**33.3**	**66.7**	**28.6**	**14.3**	**23.8**	**28.6**	**33.3**	**19.0**	**19.0**	**14.3**	**14.3**

Twenty-two studies reported PCPs with an intra-operative phase, consisting of a median of 3 individual components. One hundred and ten intra-operative component interventions were mapped to 12 intra-operative intervention domains (Table 3). Commonly covered domains across PCPs intra-operatively were prescriptive surgical strategy (*n* = 13, 59.1%), prescriptive anaesthetic strategy (*n* = 10, 45.5%), normothermia (*n* = 12, 54.5%), goal directed fluid therapy (*n* = 10, 45.5%) and analgesia (*n* = 14, 63.6%).

**Table 3:** Intra-operative intervention domains

Author	Number of component interventions	Timely surgical intervention	Senior clinical input	Antibiotic treatment	Goal directed Fluid Treatment	Normothermia	PONV	Monitoring	Prescriptive anaesthetic strategy	Prescriptive surgical strategy	Analgesia	Risk Stratification
Moller	3				*	*				*		
Huddart	6			*		*			*	*		*
Lohsiriwat	5				*	*				*	*	
Gonenc	3				*	*				*		
Wisley	2								*		*	
Tengburg	14					*	*	*		*	*	*
Shang	8					*	*		*	*	*	
Mohsina	2										*	
Burcharth	2				*				*	*		
Liska	5				*	*				*		
Lohsiriwat (2019)	8				*	*				*	*	
Aggarwal	3					*					*	
Peden	14	*	*	*	*	*	*	*	*		*	*
Saurabh	2								*		*	
Vinas	6				*				*	*		
Ong	1		*									
Sharma	6				*	*			*	*	*	
Pranavi	2										*	
Timan	1								*			
Ceresoli	12				*	*			*	*	*	
Pathrikar	2										*	
Trangbæk	3									*	*	*
Domain Coverage	110	4.5	9.1	9.1	45.5	54.5	13.6	9.1	45.5	59.1	63.6	18.2

Twenty-five studies reported PCPs with a post-operative phase, consisting of a median of 8 components (Table 4). A total of 191 individual component interventions were identified and mapped to 18 post-operative intervention domains. The most commonly employed domain interventions across PCPs were early nutrition, early mobilisation, early removal of drains and analgesia.

**Table 4:** Post-operative intervention domains

Author	Number of component interventions	Monitoring	Analgesia	PONV	Normothermia	Medical Optimisation	Early nutrition	Chest Physiotherapy	Early Mobilisation	Early removal of drains	Fluid therapy	Discharge/ Follow up criteria	Antibiotic treatment	Thrombo-prophylaxis	Critical care/ Dedicated observational ward	Review and escalation policies	Patient Counselling	Stress ulceration prophylaxis	Optimisation of Gastrointestinal function
Moller	17	*	*			*	*	*	*	*	*					*			
Huddart	1											*			*				
Lohsiriwat	9		*				*		*	*	*	*							
Gonenc	5						*			*									
Vester-Andersen	16	*									*								
Wisley	5			*			*		*	*									*
Tengburg	2		*				*		*						*				
Shang	7		*				*		*	*									
Mohsina	8		*	*			*	*	*	*								*	
Burcharth	12	*	*			*	*		*	*						*	*		
Liska	11						*	*	*	*				*					*
Lohsiriwat (2019)	3						*		*										
Aggarwal	8		*				*		*	*									
Peden	11	*	*	*	*	*	*	*					*	*	*	*			
Saurabh	10		*				*		*	*		*	*						
Vinas	10		*				*		*	*		*	*	*				*	
Fischer	5		*				*		*	*				*					
Ong	2					*									*				
Sharma	9		*	*			*		*	*		*							
Masood	5						*			*			*		*				
Boden	3							*	*								*		
Pranavi	8		*				*		*	*			*					*	
Timan	4	*	*								*					*			
Ceresoli	6						*		*	*									
Pathrikar	9		*				*		*	*			*					*	
Trangbæk	5		*							*					*				
Domain Coverage	191	20.0	64.0	16.0	4.0	16.0	80.0	20.0	72.0	72.0	16.0	20.0	24.0	16.0	24.0	16.0	8.0	16.0	8.0

### PCPs tidier checklist

The intervention characteristics of PCPs according to the TIDieR framework are outlined in Table 5. The majority of studies (*n* = 20, 66.6%) did not report the TIDieR framework items, with thirteen studies reporting less than 50% of all items. Three studies reported 90% of the items within the TIDiER framework; reporting on all components of the PCPs intervention, apart from the item on modifications. There was no in-depth detail provided across all PCPs regarding the component intervention delivered, with no data provided on component interventions in specific patient or clinical groups. The PCP designed for use by Burcharth et al. was designed specifically in keeping with the TIDiER framework [42]. The commonest TIDiER item reported across all studies was Item 2: why to describe the rationale, theory, or goal of the elements essential to the intervention. Poorly reported domains included Item 5: Who provided the interventions (*n* = 8, 30.8%), Item 7: Where (*n* = 7, 26.9%), and Item 9: Tailoring (*n* = 5, 19.2%). There was a failure to report Item 10: Modifications across all studies.

**Table 5:** TiDiER checklist

Tidier Domain	Name	Why	What		Who Provided	How	Where	When and How Much	Tailoring	Modifications	How well		Overall Domain Coverage (%)
			Materials	Procedures							Planned	Actual	
**Author**													
Moller	No	Yes	No	Yes	Yes	Yes	Yes	Yes	No	No	No	Yes	58.3
Doyle/Ebm/Huddart	ELQuiP	Yes	No	Yes	No	Yes	Yes	Yes	Yes	No	No	Yes	66.7
Lohsiriwat	No	Yes	Yes	Yes	No	Yes	No	Yes	No	No	No	No	41.7
Gonenc	No	Yes	No	Yes	No	Yes	No	Yes	No	No	No	No	33.3
Vester-Andersen	Incare	Yes	Yes	Yes	Yes	Yes	Yes	Yes	Yes	No	Yes	Yes	91.7
Wisley	No	Yes	No	No	No	No	No	No	No	No	No	No	8.3
Tengburg	No	Yes	Yes	Yes	Yes	Yes	Yes	Yes	Yes	No	No	No	66.7
Shang	No	Yes	Yes	Yes	No	No	No	Yes	No	No	No	No	50.0
Mohsina	No	Yes	No	Yes	No	Yes	No	Yes	No	No	No	No	33.3
Burcharth	OMEGA	Yes	Yes	Yes	Yes	Yes	Yes	Yes	Yes	No	Yes	Yes	91.7
Liska	No	Yes	No	Yes	No	Yes	No	Yes	No	No	No	Yes	41.7
Lohsiriwat (2019)	No	Yes	Yes	Yes	No	No	No	No	No	No	No	No	25.0
Aggarwal	No	Yes	Yes	Yes	Yes	Yes	No	Yes	No	No	No	No	50.0
Peden	EPOCH	Yes	Yes	Yes	No	No	No		No	No	Yes	Yes	50.0
Saurabh	No	Yes	No	Yes	No	No	No	Yes	No	No	No	No	25.0
Vinas	No	Yes	No	Yes	No	Yes	No	Yes	No	No	No	Yes	41.7
Fischer	No	Yes	No	Yes	No	No	No	No	No	No	No	Yes	25.0
Ong	No	Yes	No	Yes	Yes	No	No	No	No	No	No	Yes	25.0
Sharma	No	Yes	Yes	Yes	No	Yes	No	No	No	No	No	No	33.3
Masood	No	Yes	Yes	Yes	No	Yes	No	Yes	No	No	No	No	41.7
Boden	ICEAGE	Yes	Yes	Yes	Yes	Yes	Yes	Yes	Yes	N/A	Yes	Yes	91.7
Pranavi	No	Yes	Yes	Yes	No	Yes	No	Yes	No	No	No	No	41.7
Timan	SMASH	Yes	No	Yes	Yes	No	Yes	Yes	No	N/A	N/A	N/A	50.0
Ceresoli	No	Yes	Yes	Yes	No	No	No	Yes	No	No	Yes	Yes	50.0
Pathrikar	No	Yes	Yes	Yes	No	Yes	No	Yes	No	No	No	No	41.7
Trangbæk	Abdominal Surgery Acute Protocol (ASAP)	Yes	Yes	No	No	No	No	No	No	No	No	No	25.0
**Individual Domain Coverage (%)**	26.9	100.0	57.7	92.3	30.8	61.5	26.9	73.1	19.2	0.0	19.2	42.3	

### PCPs reported outcomes

Seventeen studies reported on a primary outcome; with 6 studies reporting on post-operative mortality, 3 studies on length of stay (LoS), 3 studies reported on outcomes related to complications, 2 studies reported of composite post-operative outcomes, 1 study reported on compliance, 1 study reported on cost and 1 study reported on gastrointestinal function.

A total of 250 individual outcomes were extracted from 30 studies and mapped to 13 overarching categories: mortality, length of stay (LoS), readmission, reoperation, complications, gastrointestinal function, invasive tube removal, analgesic requirements, mobility, cost-effectiveness, compliance rates, post-operative treatment, recovery and function and quality of life (QoL) (Table 6). Clinical outcomes such as morbidity, mortality and LoS were most commonly reported. Outcomes relating to analgesic requirement, compliance, mobility, recovery, function and QoL were poorly reported across all studies.

**Table 6:**
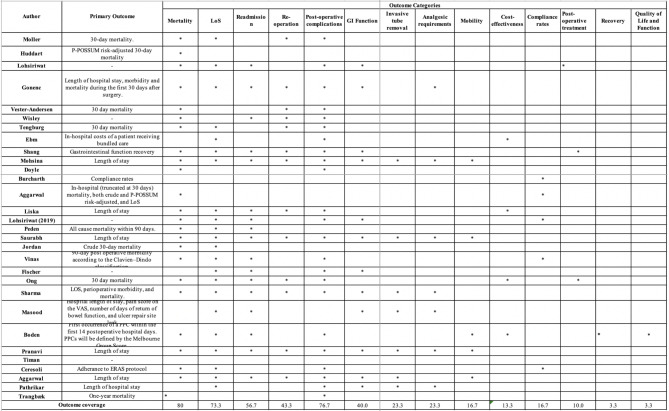
Outcome Reporting Across All Studies

Post-operative mortality was the most frequently reported outcome measure across all studies, with 24 (80%) studies reporting this outcome. However, there was significant heterogeneity in the definitions and timing in reporting this outcome measure, with 8 different definitions identified. The most commonly used definition was overall 30-day mortality, with other definitions including in-hospital and risk-adjusted mortality, as well as reporting mortality outcomes at 90 days, 180 days and 1 year post-operatively. Post-operative morbidity was reported by 23 (76.7%) studies in 27 different ways at variable timepoints ranging from 3 to 180 days post-operatively. Seven studies reported specific complications including pulmonary complications, acute kidney injury, ileus, surgical site infection, post-operative bleeding, trocar site hernia, urinary tract infections, septic shock, anastomotic leak, peritonitis and abscess. Two grading systems were identified to grade the severity of complications; the Clavien-Dindo classification and the Post-operative Morbidity Score, across 7 studies. Outcomes for the gastrointestinal function domain were reported across 12 (40%) studies using 8 different definitions. No standardised definition of gastrointestinal function was identified. Patient-reported outcomes such as recovery and function and QoL were poorly reported, with 6 identified outcome measures across 2 (6.6%) studies.

## Discussion

We highlight the heterogenous nature of current perioperative pathway design in the EmLap setting, with multiple component interventions delivered in a variable manner. Our review identified 400 individual components mapping to 24 domains, with variable quality intervention and outcome reporting as measured by the TIDiER checklist. The overall lack of intervention description and reporting for EmLap perioperative pathways limits understanding their effectiveness, implementation and generalisability. EmLap perioperative pathways consist of several interacting components, with little understanding of the underlying interaction due to the variable quality evidence base underpinning each component intervention [49, 50]. This leads to significant heterogeneity in the type of interventions employed, with this systematic review identifying 26 unique perioperative pathways. Although the interventions delivered within these pathways mapped to 24 broad overarching domains, the overall delivery and reporting of individual interventions within these domains was heterogenous and inconsistent across different pathways.

The TIDieR framework provides a standardised and robust manner to report complex interventions to enable broader adoption and implementation. However, adherence to this framework is variable in EmLap perioperative pathways. There is a significant focus on key aspects of the TIDieR framework including reporting on rationale for implementation and evaluation with reporting of key procedures outlined in 92.3% (*n* = 24) and materials required to deliver these procedures in 57.7% (*n* = 15). Despite the majority of studies reporting on key procedures and materials, these descriptions were often minimal or lacked sufficient detail, and therefore are unlikely to facilitate broader adoption or implementation. Several key details regarding intervention description and reporting are underreported, including, who delivered the intervention (*n* = 8, 30.8%), where the intervention was delivered (*n* = 7, 26.9%), tailoring of interventions (*n* = 5, 19.2%), modifications (*n* = 0, 0%), and planned and actual adherence (*n* = 5, 19.2%). These key reporting criteria are often underreported across a range of complex interventions in multiple disease settings, with the focus largely being on the actual intervention delivered. Key detail on the broader reporting standards of intervention delivery are essential for implementation of complex interventions such as a EmLap perioperative pathway, which is often delivered by several key members of the multidisciplinary team, at different timepoints and stages of the pathway, to a broad and heterogenous clinical population.

Three studies were identified to have excellent compliance with the TIDieR framework reporting. Vester-Andersen demonstrated variable compliance of 14.3–85.8% to key components of their complex intervention to improve post-operative EmLap care using intermediate care. However, when compared to standard care, the overall compliance to interventions was much higher due to the key reporting and educational components of the TIDieR framework [29]. Using a similar approach, Burcharth et al. were able to demonstrate overall compliance of 83% to 15 component interventions [42]. Boden et al. assessed the feasibility of implementing a complex intervention of intensive physiotherapy aimed at reducing postoperative pneumonia and improving physical recovery [23]. Through the use of the TIDieR framework the authors identified key interventions with poor compliance and implementation in clinical practice and the associated barriers/challenges. These three studies demonstrate the value of the TIDieR framework, using indepth intervention description and reporting in ensuring the delivery of effective and feasible interventions within the EmLap setting. Through robust and standardised reporting of interventions, complex interventions can be appropriately implemented into clinical practice. Transparent reporting is essential for pathway effectiveness research [51, 52] due to the complex nature of developing and implementing clinical pathways, which is further amplified in the emergency setting.This limits healthcare resource wastage through the early identification of undeliverable interventions and ensuring the delivery of concise, high-fidelity, clinically effective interventions within complex clinical settings.

This work contributes to the growing evidence-base in perioperative pathways in EmLap by identifying the content of these pathways and by identifying their associated reporting outcomes. However, our work is limited by the overall quality of the existing evidence-base, consisting primarily of moderately biased, non-randomised studies. We only identified ten RCTs for inclusion into this review. The disproportionate number of non-randomised studies is associated with inherent biases including selection bias and outcome reporting. This has a potential impact on determining the clinical effectiveness of the interventions and perioperative pathways reported within these studies. It is also important to note the limitations of the TIDIER checklist, as it has been designed for the generic use of intervention reporting across medicine and surgery leading to broad descriptors and the lack of thresholds to define adequate reporting.

## Conclusion

Perioperative pathways in the EmLap setting are complex interventions, with variable design and structure, spanning across the entire perioperative pathway. This review identified 26 unique pathways delivering 400 individual component interventions across 24 domains, with a variety of outcome metrics used to assess their clinical effectiveness. These pathways are multimodal, consisting of multiple component interventions. Currently, they are reported and therefore implemented in a variable manner. Future studies in EmLap perioperative pathways should ensure in depth reporting of the design and delivery of the pathway, including an in-depth description of component interventions, using existing frameworks such at the TIDIER framework. This will help identify component interventions that are valuable, effective and feasible for implementation in the EmLap setting.

## Data Availability

No datasets were generated or analysed during the current study.
